# Pretransplant and Posttransplant Alcohol Consumption and Outcomes in Kidney Transplantation: A Prospective Multicenter Cohort Study

**DOI:** 10.3389/ti.2022.10243

**Published:** 2022-05-30

**Authors:** Hee-Yeon Jung, Yena Jeon, Kyu Ha Huh, Jae Berm Park, Myung-Gyu Kim, Sik Lee, Seungyeup Han, Han Ro, Jaeseok Yang, Curie Ahn, Jang-Hee Cho, Sun-Hee Park, Yong-Lim Kim, Chan-Duck Kim

**Affiliations:** ^1^ Department of Internal Medicine, School of Medicine, Kyungpook National University, Kyungpook National University Hospital, Daegu, South Korea; ^2^ Department of Statistics, Kyungpook National University, Daegu, South Korea; ^3^ Department of Surgery, Yonsei University College of Medicine, Seoul, South Korea; ^4^ Department of Surgery, Samsung Medical Center, Sungkyunkwan University School of Medicine, Seoul, South Korea; ^5^ Department of Internal Medicine, Korea University College of Medicine, Seoul, South Korea; ^6^ Department of Internal Medicine, Chonbuk National University Hospital, Jeonju, South Korea; ^7^ Department of Internal Medicine, Keimyung University School of Medicine, Daegu, South Korea; ^8^ Department of Internal Medicine, Gil Hospital, Gachon University, Incheon, South Korea; ^9^ Department of Internal Medicine, Yonsei University College of Medicine, Seoul, South Korea; ^10^ Department of Internal Medicine, Seoul National University College of Medicine, Seoul, South Korea

**Keywords:** kidney transplantation, alcohol, all-cause mortality, biopsy-proven acute rejection, cardiovascular events, death-censored graft failure, low-density lipoprotein cholesterol, total cholesterol

## Abstract

The impact of pretransplant and posttransplant alcohol consumption on outcomes in kidney transplant recipients (KTRs) is uncertain. Self-reported alcohol consumption was obtained at the time of transplant and 2 years after transplant in a prospective cohort study. Among 907 KTRs, 368 (40.6%) were drinkers at the time of transplant. Compared to non-drinkers, alcohol consumption did not affect the risk of death-censored graft failure (DCGF), biopsy-proven acute rejection (BPAR), cardiovascular events, or all-cause mortality. Compared to persistent non-drinkers, the development of DCGF, BPAR, cardiovascular events, all-cause mortality, or posttransplant diabetes mellitus was not affected by the alcohol consumption pattern (persistent, *de novo*, or stopped drinking) over time. However, *de novo* drinkers had a significantly higher total cholesterol (*p* < 0.001) and low-density lipoprotein cholesterol levels (*p* = 0.005) compared to persistent non-drinkers 5 years after transplant, and had significantly higher total cholesterol levels (*p* = 0.002) compared to the stopped drinking group 7 years after transplant, even after adjusting for the use of lipid-lowering agents, age, sex, and body mass index. Although pretransplant and posttransplant alcohol consumption were not associated with major outcomes in KTRs during the median follow-up of 6.0 years, a new start of alcohol use after KT results in a relatively poor lipid profile.

**Clinical Trial Registration:** clinicaltrials.gov, identifier NCT02042963.

## Graphical Abstract

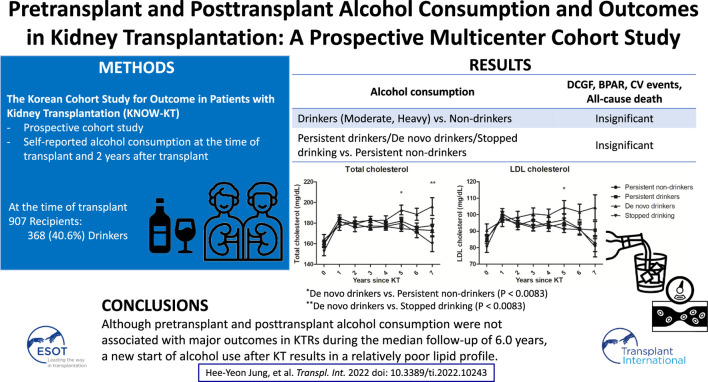

## Introduction

Though previous studies have reported that moderate alcohol consumption is associated with the improvement of some lipid profiles (1–3), as well as a reduced risk of cardiovascular events (4–6), including myocardial infarction, stroke, and heart failure, and mortality (7, 8) in the general population, recent evidence suggests that there is no safe level of moderate drinking in terms of mortality (9). However, robust evidence is lacking as to whether the potential protective effect of moderate alcohol use can be generalized to kidney transplant recipients (KTRs), or whether alcohol is an acceptable beverage for KTRs in terms of transplant outcomes. It is important to identify the effects of alcohol consumption in KTRs because transplant patients are on immunosuppressants; alcohol use may affect the metabolism of immunosuppressive agents and, thus, transplant outcomes. Alcohol metabolism by the cytochrome P450 enzyme system (CYP2E1) may be a potent enzyme inducer, and immunosuppressants are metabolized by CYP3A4; therefore, alcohol use may result in unexpected variation in immunosuppressant levels (10, 11). Moreover, KTRs have a large burden of cardiovascular complications, so it is necessary to determine the effects of alcohol consumption.

The Kidney Disease Improving Global Outcome (KDIGO) clinical practice guidelines do not provide specific guidance on alcohol consumption in KTRs (12). Surprisingly, relatively few studies have reported the effects of pretransplant (13, 14) or posttransplant (15, 16) alcohol use in KTRs, and these few have reported inconsistent results in terms of recipient mortality (13, 16). Furthermore, the impact of pretransplant and posttransplant alcohol consumption over time on major outcomes, including kidney graft survival, patient survival, biopsy-proven acute rejection (BPAR), cardiovascular events, kidney function, and glucose and lipid metabolism, has not been explored in KTRs in a prospective study design.

The present study was prospective multicenter longitudinal cohort study aiming to determine the association between pretransplant and posttransplant alcohol consumption and comprehensive outcomes in KTRs.

## Methods

### Study Participants

**FIGURE 1:**
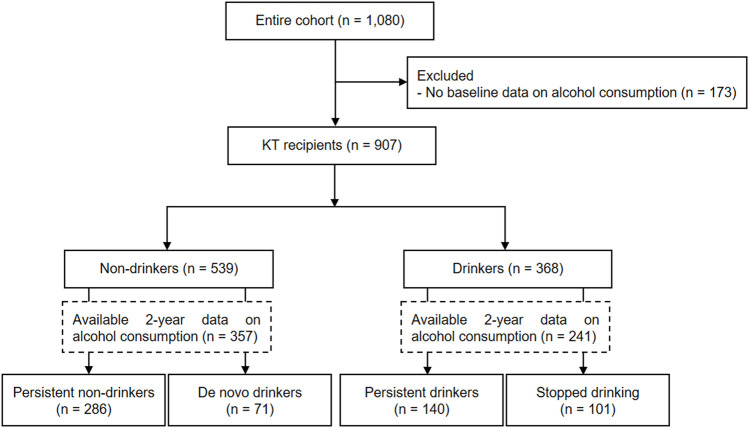
Flow chart of study inclusion.

A total of 1,080 incident KTRs were enrolled from the Korean Cohort Study for Outcome in Patients with Kidney Transplantation (KNOW-KT) between 2012 and 2016 and followed up until 2020 (clinicaltrials.gov, identifier NCT02042963). After excluding 173 KTRs who had insufficient information on baseline alcohol consumption, 907 KTRs were included in this study. Among 598 KTRs with available alcohol information 2 years after transplant, 286 (47.8%) and 140 (23.4%) KTRs remained as persistent non-drinkers and persistent drinkers, respectively, and 71 (11.9%) KTRs became *de novo* drinkers and 101 (16.9%) KTRs stopped drinking (Figure 1).

### Alcohol Consumption

Self-reported alcohol consumption was obtained from KTRs at the time of transplant and 2 years after transplant in a prospective multicenter longitudinal cohort study. Participants were asked how often they drank during the year prior to the transplant and how many drinks they drank at one time. KTRs were categorized as non-drinkers and drinkers based on baseline alcohol consumption, and alcohol consumption was categorized into two groups: moderate and heavy drinkers. The criteria for heavy drinking defined by the National Institute on Alcohol Abuse and Alcoholism (NIAAA) are as follows: for men, consuming more than four drinks on any day or more than 14 drinks per week; for women, consuming more than three drinks on any day or more than seven drinks per week (17). Pretransplant and posttransplant alcohol consumption over time was used to categorize KTRs as persistent non-drinker, persistent drinker, *de novo* drinker, and stopped drinking.

### Outcomes

Outcomes included death-censored graft failure (DCGF), biopsy-proven acute rejection (BPAR), cardiovascular events, all-cause death, estimated glomerular filtration rate (eGFR), serum creatinine, posttransplant diabetes mellitus (PTDM), and lipid profiles. DCGF was defined as dialysis or new kidney transplant. BPAR was defined as biopsy-proven acute T cell-mediated rejection or acute antibody-medicated rejection. The Modification of Diet in Renal Disease (MDRD) study equation was used to calculate the eGFR. Cardiovascular events included myocardial infarction, unstable angina, percutaneous coronary intervention, coronary artery bypass grafting, and stroke.

### Other Variables

Possible confounders for DCGF and BPAR were recipient age, donor age, recipient sex, donor sex, recipient body mass index (BMI) (18), diabetes, deceased-donor kidney transplantation (DDKT), re-transplantation, desensitization (direct crossmatch (+) plus donor-specific antibodies (+), direct crossmatch (-) plus donor-specific antibodies (+), or ABO-incompatible kidney transplantation), total number of human leukocyte antigen (HLA) mismatches, and antithymocyte globulin induction. Possible confounders for cardiovascular events and all-cause mortality were recipient age, recipient sex, recipient BMI, diabetes, hypertension, coronary artery disease, cerebrovascular disease, total cholesterol, high-density lipoprotein (HDL) cholesterol, DDKT (19), re-transplantation, desensitization, total number of HLA mismatches, antithymocyte globulin induction, use of cyclosporine or inhibitor of the mammalian target of rapamycin (sirolimus or everolimus), and steroid dose 1 year after transplantation. Possible confounders for PTDM included recipient age, recipient sex, recipient BMI, baseline HbA1c, total cholesterol, low-density lipoprotein (LDL) cholesterol, HDL cholesterol, triglycerides (TGs), re-transplantation, desensitization, total number of HLA mismatches, and antithymocyte globulin induction.

### Statistical Analysis

Continuous variables were expressed as mean ± standard deviation or a median with the interquartile range (IQR). Intergroup differences were assessed by independent sample *t*-tests, chi-squared tests, and analysis of variance as appropriate. The Cox proportional hazards model was used to analyze the association between alcohol consumption and the development of DCGF, BPAR, cardiovascular events, or all-cause death. Logistic regression analysis was used to examine the association between alcohol consumption and the development of PTDM because posttransplant diabetes mellitus was recorded by annual follow-up after kidney transplantation and the exact date and year of occurrence could not be specified. A generalized linear mixed model with random slopes was used to determine the annual change in eGFR and serum creatinine by alcohol consumption group. Analysis of variance and the general linear model were used to determine between-group differences in the annual eGFR and lipid profiles, respectively. In the case of an overall F-test *p* < 0.05 when comparing the entire group, the comparison between the two groups was confirmed by Bonferroni’s post hoc method. The post hoc *p*-value adds six comparisons at the significance level of 0.05, so if the post hoc *p*-value was <0.0083, it was considered significant. When comparing outcomes between persistent non-drinkers, persistent drinkers, *de novo* drinkers, and KTRs who stopped drinking, events that occurred within 2 years posttransplant were excluded. Statistical analyses were performed using the SAS system for Windows, version 9.4 (SAS Institute Inc., Cary, NC, United States) and R (R Foundation for Statistical Computing, Vienna, Austria; www.r-project.org). *p* < 0.05 was considered significant.

## Results

### Baseline Characteristics

Table 1 shows the baseline characteristics according to baseline alcohol consumption. Among 907 eligible KTRs, 539 (59.4%) were non-drinkers and 368 (40.6%) were drinkers at the time of transplantation. Among the drinkers, 77.4% were moderate drinkers and 22.6% were heavy drinkers. Drinkers were significantly younger, tended to be male, had a higher proportion of diabetes and a lower proportion of coronary artery disease, were less likely to have received desensitization, and had lower total cholesterol levels compared to non-drinkers. We observed no significant differences in immunosuppressant types, doses, and drug concentrations at the time of discharge and 1 year after kidney transplantation between non-drinkers, moderate drinkers, and heavy drinkers.

**TABLE 1:** Baseline characteristics.

	Non-drinkers (*n* = 539)	Drinkers (*n* = 368)	*p*-value	Non-drinkers (*n* = 539)	Moderate drinkers (*n* = 285)	Heavy drinkers^d^ (*n* = 83)	*p*-value
Age, years	46.5 ± 10.7	43.2 ± 11.7	<0.001	46.5 ± 10.7^c^	44.1 ± 11.3^b^	40.4 ± 12.5^a^	<0.001
Sex, male	317 (58.8)	269 (73.1)	<0.001	317 (58.8)	205 (71.9)	64 (77.1)	<0.001
BMI, kg/m^2^	23.1 ± 3.6	22.7 ± 3.4	0.122	23.1 ± 3.6	22.7 ± 3.4	22.7 ± 3.4	0.303
Diabetes	147 (27.3)	71 (19.3)	0.006	147 (27.3)	57 (20.0)	14 (16.9)	0.019
Hypertension	498 (92.4)	336 (91.3)	0.554	498 (92.4)	262 (91.9)	74 (89.2)	0.601
Coronary artery disease	41 (8.0)	15 (4.3)	0.029	41 (8.0)	12 (4.4)	3 (3.8)	0.090
Cerebrovascular disease	20 (3.9)	7 (2.0)	0.113	20 (3.9)	6 (2.2)	1 (1.3)	0.261
Donor type							
Living	447 (82.9)	299 (81.3)	0.515	447 (82.9)	225 (79.0)	74 (89.2)	0.082
Deceased	92 (17.1)	69 (18.8)		92 (17.1)	60 (21.1)	9 (10.8)	
Total number of HLA mismatches, median (IQR)	3.0 (1.0–3.0)	3.0 (2.0–3.5)	0.319	3.0 (1.0–3.0)	3.0 (2.0–3.0)	3.0 (2.0–4.0)	0.503
Re-transplantation	39 (7.2)	21 (5.7)	0.363	39 (7.2)	18 (6.3)	3 (3.6)	0.452
Desensitization	154 (28.6)	81 (22.0)	0.027	154 (28.6)	58 (20.4)	23 (27.7)	0.035
Induction therapy							
IL-2RB	491 (91.1)	338 (91.9)	0.691	491 (91.1)	263 (92.3)	75 (90.4)	0.795
ATG	48 (8.9)	30 (8.2)		48 (8.9)	22 (7.7)	8 (9.6)	
Immunosuppressants at discharge							
Tacrolimus	511 (94.8)	338 (91.9)	0.074	511 (94.8)	261 (91.6)	77 (92.8)	0.188
Tacrolimus dose, mg/day	5.0 (3.0–8.0)	5.5 (4.0–8.0)	0.434	5.0 (3.0–8.0)	5.5 (4.0–8.0)	6.0 (3.5–9.0)	0.707
Tacrolimus dose/kg	0.10 ± 0.07	0.10 ± 0.06	0.711	0.10 ± 0.07	0.10 ± 0.07	0.10 ± 0.06	0.933
Cyclosporine	26 (4.8)	26 (7.1)	0.154	26 (4.8)	20 (7.0)	6 (7.2)	0.361
Cyclosporine dose, mg/day	254.8 ± 79.7	257.7 ± 111.3	0.915	254.8 ± 79.7	266.3 ± 113.9	229.2 ± 106.6	0.712
Cyclosporine dose/kg	4.2 ± 1.6	4.2 ± 1.9	0.956	4.2 ± 1.6	4.4 ± 2.0	3.4 ± 1.5	0.466
Sirolimus	8 (3.3)	16 (4.4)	0.433	8 (3.3)	13 (4.6)	3 (3.6)	0.678
Everolimus	6 (1.1)	11 (3.0)	0.041	6 (1.1)	8 (2.8)	3 (3.6)	0.110
Everolimus dose, mg/kg	2.2 ± 0.7	1.9 ± 0.8	0.472	2.2 ± 0.7	1.6 ± 0.2	2.7 ± 1.3	0.063
Everolimus dose/kg	0.03 ± 0.01	0.03 ± 0.02	0.860	0.03 ± 0.01	0.03 ± 0.01	0.05 ± 0.03	0.249
Steroid	535 (99.3)	366 (99.5)	0.717	535 (99.3)	283 (99.3)	83 (100.0)	1.000
Steroid dose, mg/day	16.0 (10.0–20.0)	16.0 (20.0–24.0)	0.054	16.0 (10.0–20.0)	16.0 (10.0–24.0)	16.0 (10.0–24.0)	0.150
Immunosuppressants 1 year posttransplant							
Tacrolimus	471 (87.4)	313 (85.1)	0.314	471 (87.4)	243 (85.3)	70 (84.3)	0.589
Tacrolimus dose, mg/day	3.0 (2.0–5.0)	3.0 (2.0–4.5)	0.918	3.0 (2.0–5.0)	3.0 (2.0–4.5)	3.0 (2.0–5.0)	0.726
Tacrolimus dose/kg	0.06 ± 0.04	0.06 ± 0.04	0.728	0.06 ± 0.04	0.06 ± 0.04	0.06 ± 0.04	0.908
Tacrolimus trough levels, ng/ml	6.2 ± 2.4	5.9 ± 2.2	0.089	6.2 ± 2.4	5.8 ± 2.1	6.1 ± 2.5	0.145
Cyclosporine	24 (4.5)	19 (5.2)	0.621	24 (4.5)	15 (5.3)	4 (4.8)	0.873
Cyclosporine dose, mg/day	138.5 ± 74.8	125.0 ± 55.9	0.515	138.5 ± 74.8	116.7 ± 59.5	156.3 ± 23.9	0.799
Cyclosporine dose/kg	2.2 ± 1.4	2.0 ± 0.9	0.625	2.2 ± 1.4	1.9 ± 1.0	2.3 ± 0.4	0.536
Cyclosporine trough levels, ng/ml	103.3 ± 62.8	94.7 ± 42.5	0.615	103.3 ± 62.8	87.9 ± 41.5	120.5 ± 41.1	0.508
Sirolimus	28 (5.2)	27 (7.3)	0.184	28 (5.2)	22 (7.2)	5 (6.0)	0.352
Everolimus	10 (1.9)	7 (1.9)	0.959	10 (1.9)	4 (1.4)	3 (3.6)	0.425
Steroid	458 (85.0)	316 (85.9)	0.708	458 (85.0)	247 (86.7)	69 (83.1)	0.676
Steroid dose, mg/day	5.0 (5.0–6.0)	5.0 (5.0–10.0)	0.056	5.0 (5.0–6.0)	5.0 (5.0–10.0)	5.0 (4.0–10.0)	0.072
Total cholesterol, mg/dl	156.3 ± 41.1	150.7 ± 41.3	0.048	156.3 ± 41.1	152.0 ± 40.7	146.3 ± 43.2	0.076
LDL cholesterol, mg/dl	84.7 ± 31.4	81.1 ± 30.2	0.098	84.7 ± 31.4	82.2 ± 30.1	77.5 ± 30.6	0.127
HDL cholesterol, mg/dl	45.4 ± 16.7	46.1 ± 17.1	0.561	45.4 ± 16.7	46.2 ± 16.2	45.7 ± 19.9	0.821
TGs, mg/dl	124.2 ± 82.2	124.4 ± 89.8	0.969	124.2 ± 82.2	122.4 ± 89.5	131.3 ± 91.1	0.709

Post hoc by Bonferroni’s method (a < b < c). ^d^The criteria for heavy drinking defined by the National Institute on Alcohol Abuse and Alcoholism are as follows: for men, consuming more than 4 drinks on any day or more than 14 drinks per week; for women, consuming more than 3 drinks on any day or more than 7 drinks per week.

Values are given as the mean ± standard deviation or *n* (%) unless otherwise noted.

ATG, antithymocyte globulin; BMI, body mass index; HDL, high-density lipoprotein; HLA, human leukocyte antigen; IL-2RB, interleukin-2 receptor blocker; LDL, low-density lipoprotein; TGs, triglycerides.

### Alcohol Consumption and Major Outcomes

During a median follow-up of 6.0 (IQR 4.9–7.0), 5.9 (IQR 4.7–7.0), 6.0 (IQR 5.0–7.0), and 6.1 (IQR 5.1–7.0) years, 46 DCGFs, 102 BPARs, 36 cardiovascular events, and 21 all-cause deaths occurred, respectively. Multivariate Cox regression analysis demonstrated no significant differences in the risk of DCGF, BPAR, cardiovascular events, or all-cause death between non-drinkers and drinkers (Table 2). Comparing non-drinkers, moderate drinkers, and heavy drinkers also showed consistent results. No significant differences in the risk of DCGF, BPAR, cardiovascular events, or all-cause death were observed between persistent non-drinkers and persistent drinkers, between persistent non-drinkers and *de novo* drinkers, or between persistent non-drinkers and KTRs who stopped drinking (Table 3).

**TABLE 2:** Adjusted hazard ratios (aHRs) for death-censored graft failure (DCGF), biopsy-proven acute rejection (BPAR), cardiovascular events, and all-cause death based on pretransplant alcohol consumption.

	DCGF		BPAR		Cardiovascular events		All-cause death	
Alcohol consumption^a^	aHR^b^ (95% CI)	*p*-value	aHR^b^ (95% CI)	*p*-value	aHR^c^ (95% CI)	*p*-value	aHR^c^ (95% CI)	*p*-value
Drinker vs. Non-drinker	0.95 (0.52–1.75)	0.875	1.03 (0.68–1.54)	0.898	0.54 (0.22–1.31)	0.713	1.39 (0.43–4.43)	0.581
Moderate drinker vs. Non-drinker	0.87 (0.44–1.70)	0.680	1.06 (0.68–1.64)	0.805	0.56 (0.22–1.45)	0.233	1.57 (0.49–5.02)	0.444
Heavy drinker vs. Non-drinker	1.37 (0.51–3.69)	0.533	1.05 (0.51–2.17)	0.896	0.42 (0.05–3.28)	0.410	0.00	0.999
Heavy drinker vs. Moderate drinker	1.30 (0.43–3.90)	0.641	0.94 (0.44–2.04)	0.884	0.99 (0.10–10.03)	0.991	0.00	0.997

a The criteria for heavy drinking defined by the National Institute on Alcohol Abuse and Alcoholism are as follows: for men, consuming more than 4 drinks on any day or more than 14 drinks per week; for women, consuming more than 3 drinks on any day or more than 7 drinks per week.

b Adjusted for recipient age, donor age, recipient sex, donor sex, recipient body mass index, diabetes, deceased-donor kidney transplantation, re-transplantation, desensitization, total number of human leukocyte antigen mismatches, and antithymocyte globulin induction.

c Adjusted for recipient age, recipient sex, recipient body mass index, diabetes, hypertension, coronary artery disease, cerebrovascular disease, total cholesterol, high-density lipoprotein cholesterol, deceased-donor kidney transplantation, re-transplantation, desensitization, total number of human leukocyte antigen mismatches, antithymocyte globulin induction, use of cyclosporine, sirolimus, or everolimus 1 year posttransplant, and steroid dose 1 year posttransplant.

CI, confidence interval.

**TABLE 3:** Adjusted hazard ratios (aHRs) for death-censored graft failure (DCGF), biopsy-proven acute rejection (BPAR), cardiovascular events, and all-cause death based on pretransplant and posttransplant alcohol consumption.

	DCGF		BPAR		Cardiovascular events		All-cause death	
	aHR^a^ (95% CI)	*p*-value	aHR^a^ (95% CI)	*p*-value	aHR^b^ (95% CI)	*p*-value	aHR^b^ (95% CI)	*p*-value
Persistent non-drinkers	1.00 (Ref)		1.00 (Ref)		1.00 (Ref)		1.00 (Ref)	
Persistent drinkers	0.56 (0.18–71.74)	0.315	0.72 (0.12–4.20)	0.711	0.00	0.996	1.07 (0.09–13.25)	0.960
*De novo* drinkers	0.59 (0.13–2.64)	0.488	0.87 (0.09–8.31)	0.900	3.95 (0.69–22.47)	0.122	1.69 (0.12–24.56)	0.700
Stopped drinking	0.42 (0.09–1.86)	0.251	1.24 (0.22–7.20)	0.808	0.00	0.997	2.39 (0.17–33.16)	0.515

a Adjusted for recipient age, donor age, recipient sex, donor sex, recipient body mass index, diabetes, deceased-donor kidney transplantation, re-transplantation, desensitization, total number of human leukocyte antigen mismatches, and antithymocyte globulin induction.

b Adjusted for recipient age, recipient sex, recipient body mass index, diabetes, hypertension, coronary artery disease, cerebrovascular disease, total cholesterol, high-density lipoprotein cholesterol, deceased-donor kidney transplantation, re-transplantation, desensitization, total number of human leukocyte antigen mismatches, and antithymocyte globulin induction, use of cyclosporine, sirolimus, or everolimus 1 year posttransplant, and steroid dose 1 year posttransplant.

CI, confidence interval.

**FIGURE 2:**
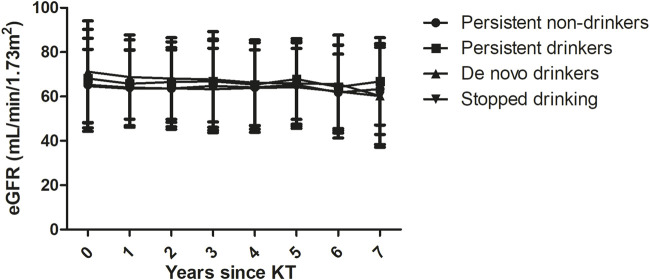
Annual estimated glomerular filtration rates (eGFRs) between groups based on alcohol consumption over time. No significant differences were observed between persistent non-drinkers, persistent drinkers, *de novo* drinkers, and the stopped drinking group.

Table 4 shows the annual changes in eGFR and serum creatinine according to pretransplant alcohol consumption. Compared to non-drinkers, no significant annual changes in eGFR and serum creatinine were observed in moderate drinkers and heavy drinkers, or when taking all drinkers. No significant differences in annual eGFR were observed between persistent non-drinkers, persistent drinkers, *de novo* drinkers, and KTRs who stopped drinking (Figure 2).

**TABLE 4:** Annual change in the estimated glomerular filtration rate (eGFR) and serum creatinine (sCr) levels according to pretransplant alcohol consumption.

Alcohol consumption group	eGFR, ml/min/1.73 m^2^/yr (95%CI)	*p*-value	sCr, mg/dl (95% CI)	*p*-value
Non-drinker	0.21 (−0.12–0.55)	Ref	−0.01 (−0.03–0.00)	Ref
Drinker	−0.19 (−0.63–0.24)	0.389	0.01 (−0.01–0.02)	0.392
Moderate drinker	−0.13 (−0.47–0.21)	0.465	0.00 (−0.01–0.02)	0.925
Heavy drinker^a^	−0.09 (−0.47–0.29)	0.655	0.01 (−0.01–0.03)	0.277

a The criteria for heavy drinking defined by the National Institute on Alcohol Abuse and Alcoholism are as follows: for men, consuming more than 4 drinks on any day or more than 14 drinks per week; for women, consuming more than 3 drinks on any day or more than 7 drinks per week.

CI, confidence interval.

### Alcohol Consumption, PTDM, and Lipid Profiles

**FIGURE 3:**
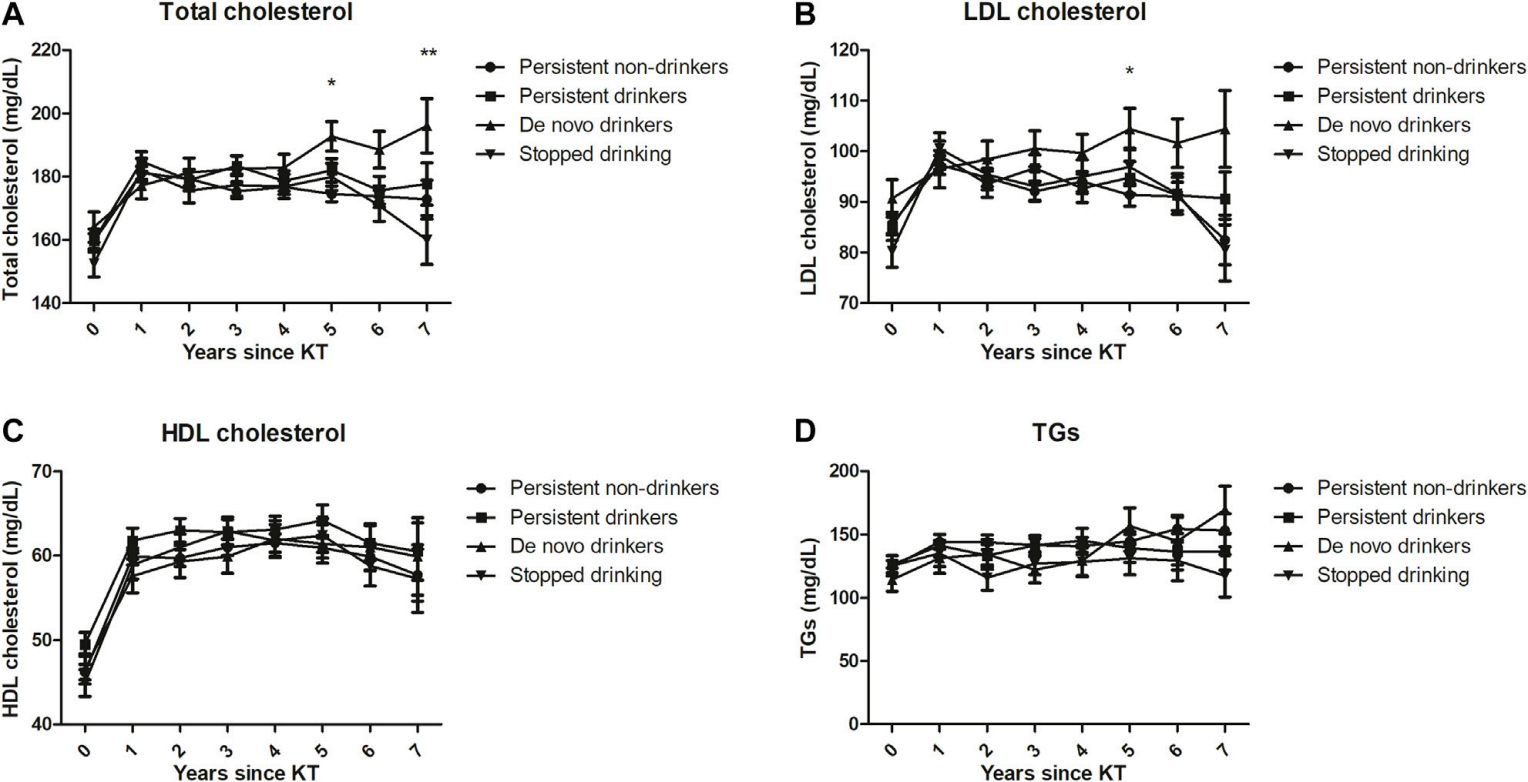
Lipid profiles [**(A)** Total cholesterol, **(B)** LDL cholesterol, **(C)** HDL cholesterol, **(D)** TGs] based on alcohol consumption over time after adjusting for the use of lipid-lowering agents, age, sex and BMI. 5 years after transplant, total cholesterol levels (*p* = 0.007) and LDL cholesterol levels (*p* = 0.044) significantly differed between the groups. In particular, total cholesterol levels (*p* < 0.001) and LDL cholesterol levels (*p* = 0.005) were significantly higher in *de novo* drinkers than in persistent non-drinkers. 7 years after transplant, total cholesterol levels significantly differed between the groups (*p* = 0.022). In particular, total cholesterol levels were significantly higher in *de novo* drinkers than in the stopped drinking group (*p* = 0.002). *indicates significant difference between *de novo* drinkers and persistent non-drinkers (*p* < 0.0083). ^**^ indicates significant difference between *de novo* drinkers and the stopped drinking group (*p* < 0.0083).

Compared to the group of persistent non-drinkers, persistent drinkers, *de novo* drinkers, and KTRs who stopped drinking were not significantly associated with the development of PTDM (Table 5). Figure 3 shows the results of the general linear model for the relationships between alcohol consumption over time and total cholesterol, LDL cholesterol, HDL cholesterol, and TGs after adjusting for the use of lipid-lowering agents, age, sex, and BMI. 5 years after transplant, there were significant differences between the groups in total cholesterol levels (*p* = 0.007) and LDL cholesterol levels (*p* = 0.044). In particular, the total cholesterol levels (192.7 ± 4.7 mg/dl vs. 174.5 ± 2.5 mg/dl, *p* < 0.001) and LDL cholesterol levels (104.4 ± 4.1 mg/dl vs. 91.4 ± 2.2 mg/dl, *p* = 0.005) were significantly higher in *de novo* drinkers than in persistent non-drinkers. 7 years after transplant, there was a significant difference between the groups in total cholesterol levels (*p* = 0.022). In particular, the total cholesterol levels were significantly higher in *de novo* drinkers than in the group that stopped drinking (196.1 ± 8.6 mg/dl vs. 160.0 ± 7.7 mg/dl, *p* = 0.002).

**TABLE 5:** Adjusted odds ratios (aORs) for posttransplant diabetes mellitus among kidney transplant recipients without pretransplant diabetes mellitus.

	aOR^a^ (95% CI)	*p*-value
Persistent non-drinkers	1.00 (Ref)	
Persistent drinkers	0.92 (0.35–2.43)	0.679
De novo drinkers	0.71 (0.20–2.50)	0.384
Stopped drinking	2.02 (0.60–6.82)	0.166

a Adjusted for recipient age, recipient sex, recipient body mass index, baseline HbA1c, total cholesterol, low-density lipoprotein cholesterol, high-density lipoprotein cholesterol, triglycerides, re-transplantation, desensitization, total number of human leukocyte antigen mismatches, and antithymocyte globulin induction.

CI, confidence interval.

## Discussion

In this prospective longitudinal cohort study, pretransplant alcohol consumption did not affect the risk of major outcomes, including DCGF, BPAR, cardiovascular events, or all-cause mortality, or annual changes in eGFR over a median follow-up of 6.0 years. The risk of major outcomes was not different according to the amount and frequency of alcohol consumption. Considering posttransplant alcohol consumption, compared to persistent non-drinkers, the development of DCGF, BPAR, cardiovascular events, all-cause mortality, or PTDM was not affected by the alcohol consumption pattern over time, including persistent drinking, *de novo* drinking, and stopped drinking. However, *de novo* drinkers had significantly higher total cholesterol and LDL cholesterol levels compared to persistent non-drinkers 5 years after transplant, and had significantly higher total cholesterol levels compared to the group that stopped drinking 7 years after transplant, even after adjusting for the use of lipid-lowering agents, age, sex, and BMI.

The prevalence of alcohol consumption at the time of transplantation (40.6%) in our study was relatively lower than the posttransplant alcohol consumption in previous kidney transplant studies (52%–52.8%) (15, 16). Compared to the prevalence of current drinkers in the general population [80%–100% in South Korean men and 60%–79.9% in South Korean women (9), 60% of Koreans drank at least once a month according to the Korea National Health and Nutrition Examination Survey 2013 (20)], considerably lower alcohol drinking rates in KTRs may reflect that the patients themselves are refraining from drinking for medical reasons or upon the advice of physicians. As for the effects of alcohol consumption on patient survival, prior studies have reported conflicting results depending on alcohol consumption before or after transplantation. One retrospective study including 425 KTRs with alcohol dependence before transplantation and 60,532 KTRs who did not use alcohol reported that pretransplant alcohol dependency is a risk factor for graft failure and patient death (13). A retrospective study of more than one million patients with kidney failure also presented that abuse of alcohol, tobacco, or drugs is associated with graft failure, but the effect of alcohol use alone was not reported (14). However, another prospective study including 600 KTRs demonstrated that moderate alcohol consumption (10–30 g/day) posttransplant is associated with a reduced risk of mortality in KTRs (16). In contrast to the results from previous studies, neither pretransplant not posttransplant alcohol use was associated with graft failure and recipient death in our study. Previous studies have not clearly identified the frequency of alcohol consumption; the various results may be due to differences in the distribution of the frequency of alcohol among heavy drinkers. Although we adjusted for considerable risk factors associated with graft failure and mortality in this study, differences in other traditional risk factors, such as smoking may affect the results.

The influence of alcohol consumption on BPAR and kidney allograft function in KTRs is still not clearly defined. Although low adherence to immunosuppressive agents has been associated with heavy drinking and dependence (21, 22), pretransplant and posttransplant alcohol consumption did not increase the risk of BPAR in KTRs in this study. This could be explained by the fact that the proportion of heavy-frequent drinkers was not high. With regard to the association between alcohol consumption and kidney function in the general population, previous studies have reported inconsistent results. No adverse outcome or protective effect of moderate alcohol consumption on kidney function has been shown in general population studies, but a decreased risk of the development of chronic kidney disease has been reported (23–26). However, other studies reported that a daily alcohol intake of 30 g or more is an independent risk factor for the development of albuminuria (27), 2 units of alcohol per day or more increases the risk of kidney failure (28), and that alcohol use has an adverse impact on kidney function (29–31). The lack of an significant association between pretransplant and posttransplant alcohol consumption and the changes in the annual kidney function in this study may also be related to the lower proportion of heavy-frequent drinkers or other stronger immunological and demographic factors than alcohol itself.

The protective effect of moderate alcohol consumption on cardiovascular disease in the general population was previously assumed to be due to alcohol-associated increases in HDL cholesterol and apolipoprotein A1 levels (32, 33), increased insulin sensitivity (34, 35), and reduced platelet aggregation (36). One kidney transplant study reported that moderate alcohol consumption (10–30 g/day) is associated with a low prevalence of PTDM (16). In contrast to our expectations and the results from previous studies, no association was found between pretransplant and posttransplant alcohol consumption and PTDM, and *de novo* drinkers had higher total cholesterol and LDL cholesterol levels than persistent non-drinkers or the stopped drinking group, even after adjusting for several related factors. Although it is difficult to determine the exact mechanism underlying this result, we cannot completely rule out the possibility that relatively higher lipid profiles in *de novo* drinkers are related to other unhealthy life style patterns that develop after transplantation, as well as the effects of alcohol itself.

In this study, we found significant differences in total cholesterol levels and LDL cholesterol levels between *de novo* drinkers and non-drinkers, but we found no significant differences between persistent drinkers and non-drinkers. This is probably due to the difference between the two groups in the amount of alcohol consumed each year after kidney transplantation. Changes in the alcohol consumption patterns of persistent drinkers were confirmed; initially, 23.6% were heavy drinkers, but this decreased to 13.6% in the second year after kidney transplantation. To clarify this, information on the amount of alcohol consumed each year after kidney transplantation will be needed in both groups. Unfortunately, in this prospective study, information on the amount of alcohol consumed each year after kidney transplantation was not obtained, so it is difficult to fully explain this with current data alone.

This study has some limitations. First, the information on alcohol use relied on self-reporting, which is susceptible to inaccurate recall or a desire to give socially acceptable answers, ultimately underestimating alcohol consumption (37–39). Second, no information was obtained regarding the type of alcohol consumed by participants. Third, because alcohol consumption was investigated based on the prior year at the time of transplant, it is possible that remote former drinkers were classified as non-drinkers. Fourth, the response rate to alcohol consumption 2 years after transplantation was 65.9%, which was not very high. Therefore, the distribution of groupings over time with alcohol consumption may not accurately reflect changes in the actual alcohol consumption pattern. Fifth, considering racial and ethnic differences in alcohol metabolism (40), the results of the present study have limited generalizability because this study included only an Asian kidney transplant population. Finally, although pretransplant and posttransplant alcohol consumption were not associated with major outcomes, including DCGF, BPAR, cardiovascular events, and all-cause death in KTRs, this study did not confirm the long-term safety of alcohol consumption in terms of other alcohol-related medical problems, such as alcohol use disorder, liver disease, or cancer (9).

Nevertheless, this study has definite strengths. Few alcohol-related research studies have been conducted in kidney transplant populations compared to the general population, and all of them have used cross-sectional alcohol consumption information. Our results were obtained from a prospective multicenter study including consecutive incident KTRs. Furthermore, this study explored both pretransplant and posttransplant alcohol use, including the amount and frequency, for the first time to evaluate the impact on adverse outcomes, which extended our knowledge. Lastly, the number of participants was considerable and the median follow-up duration considerably long.

In conclusion, although pretransplant and posttransplant alcohol consumption is not associated with major outcomes in KTRs, a new start of alcohol use after kidney transplantation results in a relatively poor lipid profile. As dyslipidemia can be associated with cardiovascular events and mortality in the long-term, the results of this study should be kept in mind when monitoring KTRs to optimize long-term transplant outcomes. Furthermore, this study did not confirm the long-term safety of alcohol in terms of other alcohol-related medical problems, such as alcohol use disorder, liver disease, or cancer, and assessment of the effects of alcohol consumption on KTRs should proceed with caution. Larger and longer-term studies will be needed to develop firm guidelines on alcohol use by KTRs.

## Data Availability

The datasets generated and analysed during the current study are not readily available because the data was collected for specific research purposes. Requests to access the datasets should be directed to So Hyeon Park, js041571@yuhs.ac.

## Abbreviations

BMI, body mass index; BPAR, biopsy-proven acute rejection; CI, confidence interval; DCGF, death-censored graft failure; eGFR, estimated glomerular filtration rate; HDL, high-density lipoprotein; HLA, human leukocyte antigen; HR, hazard ratio; IQR, interquartile range; KTR, kidney transplant recipient; LDL, low-density lipoprotein; PTDM, posttransplant diabetes mellitus; TGs, triglycerides.

## References

[1] Park H Kim K. Relationship Between Alcohol Consumption and Serum Lipid Levels in Elderly Korean Men. Arch Gerontol Geriatr (2012) 55:226–30. 10.1016/j.archger.2011.08.014 21925744

[2] Tabara Y Ueshima H Takashima N Hisamatsu T Fujiyoshi A Zaid M Mendelian Randomization Analysis in Three Japanese Populations Supports a Causal Role of Alcohol Consumption in Lowering Low-Density Lipid Cholesterol Levels and Particle Numbers. Atherosclerosis (2016) 254:242–8. 10.1016/j.atherosclerosis.2016.08.021 27575649

[3] Wakabayashi I. Difference in Sensitivities of Blood HDL Cholesterol and LDL Cholesterol Levels to Alcohol in Middle-Aged Japanese Men. Alcohol (2018) 67:45–50. 10.1016/j.alcohol.2017.08.011 29425958

[4] Leong DP Smyth A Teo KK McKee M Rangarajan S Pais P Patterns of Alcohol Consumption and Myocardial Infarction Risk: Observations from 52 Countries in the INTERHEART Case-Control Study. Circulation (2014) 130:390–8. 10.1161/circulationaha.113.007627 24928682

[5] Christensen AI Nordestgaard BG Tolstrup JS. Alcohol Intake and Risk of Ischemic and Haemorrhagic Stroke: Results from a Mendelian Randomisation Study. J Stroke (2018) 20:218–27. 10.5853/jos.2017.01466 29886720 PMC6007300

[6] Djoussé L Gaziano JM. Alcohol Consumption and Heart Failure: A Systematic Review. Curr Atheroscler Rep (2008) 10:117–20. 10.1007/s11883-008-0017-z 18417065 PMC2365733

[7] Xi B Veeranki SP Zhao M Ma C Yan Y Mi J. Relationship of Alcohol Consumption to All-Cause, Cardiovascular, and Cancer-Related Mortality in U.S. Adults. J Am Coll Cardiol (2017) 70:913–22. 10.1016/j.jacc.2017.06.054 28818200

[8] Wood AM Kaptoge S Butterworth AS Willeit P Warnakula S Bolton T Risk Thresholds for Alcohol Consumption: Combined Analysis of Individual-Participant Data for 599 912 Current Drinkers in 83 Prospective Studies. Lancet (2018) 391:1513–23. 10.1016/S0140-6736(18)30134-X 29676281 PMC5899998

[9] Griswold MG Fullman N Hawley C Arian N Zimsen SR Tymeson HD Alcohol Use and Burden for 195 Countries and Territories, 1990-2016: A Systematic Analysis for the Global Burden of Disease Study 2016. Lancet (2018) 392:1015–35. 10.1016/S0140-6736(18)31310-2 30146330 PMC6148333

[10] Iwasaki K. Metabolism of Tacrolimus (FK506) and Recent Topics in Clinical Pharmacokinetics. Drug Metab Pharmacokinet (2007) 22:328–35. 10.2133/dmpk.22.328 17965516

[11] Parker R Armstrong MJ Corbett C Day EJ Neuberger JM. Alcohol and Substance Abuse in Solid-Organ Transplant Recipients. Transplantation (2013) 96:1015–24. 10.1097/tp.0b013e31829f7579 24025323

[12] Kidney Disease: Improving Global Outcomes (KDIGO) Transplant Work Group. Special Issue: KDIGO Clinical Practice Guideline for the Care of Kidney Transplant Recipients. Am J Transpl (2009) 9(Suppl. 3):S1–S155. 10.1111/j.1600-6143.2009.02834.x 19845597

[13] Gueye AS Chelamcharla M Baird BC Nguyen C Tang H Barenbaum AL The Association between Recipient Alcohol Dependency and Long-Term Graft and Recipient Survival. Nephrol Dial Transplant (2007) 22:891–8. 10.1093/ndt/gfl689 17172252

[14] Sandhu GS Khattak M Woodward RS Hanto DW Pavlakis M Dimitri N Impact of Substance Abuse on Access to Renal Transplantation. Transplantation (2011) 91:86–93. 10.1097/tp.0b013e3181fc8903 20966832

[15] Fierz K Steiger J Denhaerynck K Dobbels F Bock A De Geest S. Prevalence, Severity and Correlates of Alcohol Use in Adult Renal Transplant Recipients. Clin Transpl (2006) 20:171–8. 10.1111/j.1399-0012.2005.00460.x 16640523

[16] Zelle DM Agarwal PK Ramirez JLP van der Heide JJH Corpeleijn E Gans ROB Alcohol Consumption, New Onset of Diabetes After Transplantation, and All-Cause Mortality in Renal Transplant Recipients. Transplantation (2011) 92:203–9. 10.1097/tp.0b013e318222ca10 21685828

[17] NIAAA. Drinking Levels Defined (2021). Available at: https://www.niaaa.nih.gov/alcohol-health/overview-alcohol consumption/moderate-binge-drinking (Accessed October 29, 2021).

[18] Curran SP Famure O Li Y Kim SJ. Increased Recipient Body Mass Index Is Associated with Acute Rejection and Other Adverse Outcomes After Kidney Transplantation. Transplantation (2014) 97:64–70. 10.1097/tp.0b013e3182a688a4 24056619

[19] Devine PA Courtney AE Maxwell AP. Cardiovascular Risk in Renal Transplant Recipients. J Nephrol (2019) 32:389–99. 10.1007/s40620-018-0549-4 30406606 PMC6482292

[20] Ministry of Health and Welfare, Korea Centers for Disease Control and Prevention. Korea Health Statistics 2013: Korea National Health and Nutrition Examination Survey (KNHANES VI-1). Cheongju: Korea Centers for Disease Control and Prevention (2014).

[21] Shapiro PA Williams DL Foray AT Gelman IS Wukich N Sciacca R. Psychosocial Evaluation and Prediction of Compliance Problems and Morbidity After Heart Transplantation. Transplantation (1995) 60:1462–6. 10.1097/00007890-199560120-00016 8545875

[22] Beresford TP Schwartz J Wilson D Merion R Lucey MR. The Short-Term Psychological Health of Alcoholic and Non-Alcoholic Liver Transplant Recipients. Alcohol. Clin Exp Res (1992) 16:996–1000. 10.1111/j.1530-0277.1992.tb01908.x 1443442

[23] Schaeffner ES Kurth T de Jong PE Glynn RJ Buring JE Gaziano JM. Alcohol Consumption and the Risk of Renal Dysfunction in Apparently Healthy Men. Arch Intern Med (2005) 165:1048–53. 10.1001/archinte.165.9.1048 15883245

[24] Knight EL Stampfer MJ Rimm EB Hankinson SE Curhan GC. Moderate Alcohol Intake and Renal Function Decline in Women: A Prospective Study. Nephrol Dial Transplant (2003) 18:1549–54. 10.1093/ndt/gfg228 12897093

[25] Yamagata K Ishida K Sairenchi T Takahashi H Ohba S Shiigai T Risk Factors for Chronic Kidney Disease in a Community-Based Population: A 10-year Follow-Up Study. Kidney Int (2007) 71:159–66. 10.1038/sj.ki.5002017 17136030

[26] Koning SH Gansevoort RT Mukamal KJ Rimm EB Bakker SJL Joosten MM. Alcohol Consumption Is Inversely Associated with the Risk of Developing Chronic Kidney Disease. Kidney Int (2015) 87:1009–16. 10.1038/ki.2014.414 25587707

[27] White SL Polkinghorne KR Cass A Shaw JE Atkins RC Chadban SJ. Alcohol Consumption and 5-year Onset of Chronic Kidney Disease: The AusDiab Study. Nephrol Dial Transplant (2009) 24:2464–72. 10.1093/ndt/gfp114 19307230

[28] Perneger TV Whelton PK Puddey IB Klag MJ. Risk of End-Stage Renal Disease Associated with Alcohol Consumption. Am J Epidemiol (1999) 150:1275–81. 10.1093/oxfordjournals.aje.a009958 10604769

[29] Menon V Katz R Mukamal K Kestenbaum B de Boer IH Siscovick DS Alcohol Consumption and Kidney Function Decline in the Elderly: Alcohol and Kidney Disease. Nephrol Dial Transplant (2010) 25:3301–7. 10.1093/ndt/gfq188 20400446 PMC2948837

[30] Bundy JD Bazzano LA Xie D Cohan J Dolata J Fink JC Self-Reported Tobacco, Alcohol, and Illicit Drug Use and Progression of Chronic Kidney Disease. Clin J Am Soc Nephrol (2018) 13:993–1001. 10.2215/cjn.11121017 29880471 PMC6032576

[31] Joo YS Koh H Nam KH Lee S Kim J Lee C Alcohol Consumption and Progression of Chronic Kidney Disease: Results from the Korean Cohort Study for Outcome in Patients with Chronic Kidney Disease. Mayo Clinic Proc (2020) 95:293–305. 10.1016/j.mayocp.2019.06.014 31883696

[32] Gaziano JM Buring JE Breslow JL Goldhaber SZ Rosner B VanDenburgh M Moderate Alcohol Intake, Increased Levels of High-Density Lipoprotein and its Subfractions, and Decreased Risk of Myocardial Infarction. N Engl J Med (1993) 329:1829–34. 10.1056/nejm199312163292501 8247033

[33] Chung B-H Doran S Liang P Osterlund L Cho BS Oster RA Alcohol-Mediated Enhancement of Postprandial Lipemia: A Contributing Factor to an Increase in Plasma HDL and a Decrease in Risk of Cardiovascular Disease. Am J Clin Nutr (2003) 78:391–9. 10.1093/ajcn/78.3.391 12936920

[34] Davies MJ Baer DJ Judd JT Brown ED Campbell WS Taylor PR. Effects of Moderate Alcohol Intake on Fasting Insulin and Glucose Concentrations and Insulin Sensitivity in Postmenopausal Women: A Randomized Controlled Trial. JAMA (2002) 287:2559–62. 10.1001/jama.287.19.2559 12020337

[35] Kiechl S Willeit J Poewe W Egger G Oberhollenzer F Muggeo M Insulin Sensitivity and Regular Alcohol Consumption: Large, Prospective, Cross Sectional Population Study (Bruneck Study). BMJ (1996) 313:1040–4. 10.1136/bmj.313.7064.1040 8898593 PMC2352363

[36] Renaud SC Beswick AD Fehily AM Sharp DS Elwood PC. Alcohol and Platelet Aggregation: the Caerphilly Prospective Heart Disease Study. Am J Clin Nutr (1992) 55:1012–7. 10.1093/ajcn/55.5.1012 1570795

[37] Gilligan C Anderson KG Ladd BO Yong YM David M. Inaccuracies in Survey Reporting of Alcohol Consumption. BMC Public Health (2019) 19:1639. 10.1186/s12889-019-7987-3 31805923 PMC6896737

[38] Gual A Ángel Arbesú J Zarco J Balcells-Oliveró Md. l. M López-Pelayo H Miquel L Risky Drinkers Underestimate Their Own Alcohol Consumption. Alcohol Alcohol (2017) 52:516–7. 10.1093/alcalc/agx029 28498886

[39] Livingston M Callinan S. Underreporting in Alcohol Surveys: Whose Drinking Is Underestimated? J Stud Alcohol Drugs (2015) 76:158–64. 10.15288/jsad.2015.76.158 25486405

[40] Edenberg HJ. The Genetics of Alcohol Metabolism: Role of Alcohol Dehydrogenase and Aldehyde Dehydrogenase Variants. Alcohol Res Health (2007) 30:5–13. 17718394 PMC3860432

[41] Yang J Lee J Huh KH Park JB Cho J-H Lee S KNOW-KT (Korean Cohort Study for Outcome in Patients with Kidney Transplantation: A 9-year Longitudinal Cohort Study): Study Rationale and Methodology. BMC Nephrol (2014) 15:77. 10.1186/1471-2369-15-77 24884405 PMC4022441

